# Perceived Environmental Factors Associated with Physical Activity among Normal-Weight and Overweight Japanese Men

**DOI:** 10.3390/ijerph8040931

**Published:** 2011-03-28

**Authors:** Yung Liao, Kazuhiro Harada, Ai Shibata, Kaori Ishii, Koichiro Oka, Yoshio Nakamura, Shigeru Inoue, Teruichi Shimomitsu

**Affiliations:** 1 Graduate School of Sport Sciences, Waseda University, Nakamura Lab. 2-579-15 Mikajima Tokorozawa, Saitama 359-1192, Japan; E-Mail: harada-ka@fuji.waseda.jp (K.H.); 2 Japan Society for the Promotion of Science, Sumitomo-Ichibancho Bldg., 6 Ichibancho, Chiyoda-ku, Tokyo 102-8471, Japan; 3 Faculty of Sport Sciences, Waseda University, 2-579-15 Mikajima Tokorozawa, Saitama 359-1192, Japan; E-Mails: aishibata@aoni.waseda.jp (A.S.); ishiikaori@aoni.waseda.jp (K.I.); koka@waseda.jp (K.O.); nakamura@waseda.jp (Y.N.); 4 Department of Preventive Medicine and Public Health, Tokyo Medical University, 6-1-1, Shinjuku-ku, Tokyo 160-8402, Japan; E-Mails: inoue@tokyo-med.ac.jp (S.I.); tshimo@tokyo-med.ac.jp (T.S.)

**Keywords:** BMI, overweight, moderator, perceived environment, walking, moderate-to-vigorous physical activity, physical activity recommendation, Japan

## Abstract

Although it is crucial to examine the environmental correlates of physical activity (PA) for developing more effective interventions for overweight populations, limited studies have investigated differences in the environmental correlates on body mass index (BMI). The purpose of the present study was to examine the perceived environmental correlates of PA among normal-weight and overweight Japanese men. Data were analyzed for 1,420 men (aged 44.4 ± 8.3 years), who responded to an internet-based cross-sectional survey of answering the short version of the International Physical Activity Questionnaire and its Environment Module. Binary logistic regression analyses were utilized to examine the environmental factors associated with meeting the PA recommendation (150 minutes/week) between the normal-weight and overweight men. After adjusting for socio-demographic variables, common and different environmental correlates of PA were observed among normal-weight and overweight men. Furthermore, significant interactions regarding PA were observed between BMI status and two environmental correlates: access to public transportation (*P* = 0.03) and crime safety during the day (*P* = 0.01). The results indicated that BMI status is a potential moderator between perceived environmental factors and PA and suggested that different environmental intervention approaches should be developed for overweight populations.

## 1. Introduction

Overweight and obesity are associated with an increased risk of morbidity from chronic diseases, as well as with higher health-care costs and lower quality of life [1–4]. An increasing prevalence of obesity has been reported in Western countries, and in those countries, the groups with higher risk of obesity varied by age, gender, and race/ethnicity [5–7]. Compared to the U.S. (where the prevalence of obesity is approximately 30%), the prevalence of obesity in Japan is much lower (approximately 3%) and has changed little during the last 40 years [8,9]. However, more recently, the prevalence of overweight adults in Japan has grown to 28.6% in men and 20.6% in women, and men aged 40–49 years had the highest percentage (35.9%) [10]. Therefore, with regard to the obesity epidemic, identifying effective, population-based strategies for preventing weight gain would be a public health priority, not only in Western countries, but also in Japan.

Numerous longitudinal and cross-sectional studies have shown that engaging in physical activity (PA) is beneficial for the prevention of obesity and overweight [11–16]. Based on these findings, the World Health Organization has recommended engaging almost daily in at least 30 minutes of moderate-intensity PA for the prevention of obesity and other chronic diseases [5,17]. Despite such a benefit, overweight and obese individuals spent less time on PA and were less likely than normal-weight individuals to meet the minimum recommended level of PA [moderate-to-vigorous PA (MVPA) at least 30 minutes per day, 5 or more days per week; ≥150 minutes per week] [12–16]. Therefore, developing effective strategies to promote PA to overweight and obese subgroups is needed to prevent further increases in the obesity rate among populations.

A better understanding of factors associated with PA is critical in designing relevant policies and effective interventions. From an ecological perspective, the manipulation of environmental attributes would be expected to provide a long-term impact on the PA of an associated population [18,19]. In this context, the association between environmental factors and PA behaviors has been reported in many countries [20]. However, many of these previous studies have been conducted in general populations [20,21]. Recent studies have suggested that environmental factors associated with PA differ between socio-demographic subgroups, such as men and women [22,23], older and younger adults [24], African-American and white adults [25], and driving and non-driving rural women [26]. In addition to these differences, BMI status has been also suggested as a potential moderator for the correlates of PA [27]. It is important to examine the factors associated with engaging in PA in the overweight subgroup to develop more tailored intervention strategies. However, to date only a Portuguese study has examined the environmental dimensions associated with meeting the PA recommendation among overweight/obese women [22], although previous studies have consistently observed gender differences in environmental correlates [22,28]. Thus, the present study examined the differences in perceived environmental factors associated with PA among normal-weight and overweight Japanese men.

## 2. Methods

### 2.1. Participants

An internet-based cross-sectional survey was conducted in January 2009 by a Japanese internet research service organization, which listed approximately 264,000 voluntarily registered subjects across Japan and their detailed socio-demographic attributes. Thus, the organization could access data from the targeted group on the basis of the requirements of each survey. In the current study, the sample size and personal attributes of the targeted group was set as follows: (1) approximately 3,000 adults aged between 30 and 59 years. (2) 500 men and 500 women in each age group (aged 30–39, 40–49, and 50–59 years). A total of 9,418 potential respondents, aged 30–59 years, were randomly selected from the database and invited to attend this internet-based survey via email (final respondents: 3,000 adults; response rate: 31.9%). The email invitations included the URL for access to this survey, and the potential respondents could log in using their own ID and password to answer the questionnaire voluntarily. The present study received prior approval from the Ethics Committee of the Faculty of Sports Sciences, Waseda University, Japan.

### 2.2. Measures

***BMI:*** Self-reported height and weight were used to calculate the body mass index (BMI; body weight in kilograms divided by the square of height in meters). The participants were classified as normal-weight men (BMI < 25) and overweight men (BMI ≥ 25) in the present study.

***Physical activity:*** Physical activity was measured by the self-administered, short version of the International Physical Activity Questionnaire (IPAQ-SV), which was recommended for the national prevalence studies [29]. IPAQ-SV, which includes seven items, was used to measure the frequency and duration of vigorous-intensity PA, moderate-intensity PA, and walking level for young and middle-aged adults (15–69 years). The test-retest reliability (r = 0.72–0.93) and criterion validity (r = 0.39) of the Japanese version of the IPAQ-SV are good and acceptable [30]. The total minutes of each PA category in a week were first computed. In the present study, two independent variables, the total minutes of walking and MVPA (excluding walking), were calculated. MVPA (excluding walking) was computed by summing the minutes per week of moderate- and vigorous-intensity PA time in the IPAQ-SV. Both walking and MVPA excluding walking were dichotomized at 150 minutes or more per week according to the public health PA recommendation [31]. In each variable, the respondents could be categorized into two groups: either meeting the recommended level or not.

***Perceived environmental factors:*** The Japanese version of the International Physical Activity Questionnaire-Environmental Module (IPAQ-E) was utilized to measure the perceived environmental factors associated with PA. The IPAQ-E questionnaire was originally developed by the International Physical Activity Prevalence Study (IPS), has been used in several countries, and has shown good reliability [18,32–34]. This self-administered questionnaire consists of three sets of items, which include seven core items, four recommended items, and six optional items [35]. In this study, all 17 items were included using a 4-point Likert scale (strongly agree, somewhat agree, somewhat disagree, and strongly disagree), with the exception of the following two questions: (1) *What is the main type of housing in your neighborhood*? For this question, the five options were detached single-family housing; apartments with 2–3 stories; mix of single-family housing and apartments with 2–3 stories; condos with 4–12 stories; and condos with >13 stories. (2) *How many household cars or auto bikes are there at your household?* This question was open ended.

For the analyses, similar to previous studies [18,36], the 17 environmental variables were converted into binary items. Residential density was divided into “detached single-family housing” and “others”, and having household car or auto bikes was classified into “0” and “>0”. Other items were categorized as “agree” (strongly agree and somewhat agree) and “disagree” (somewhat disagree and strongly disagree).

***Socio-demographic variables:*** In the present study, socio-demographic correlates included gender, age, marital status, educational level, household income, and employment status. Age was categorized as 30–39, 40–49, and 50–59 years. Marital status was classified into married and unmarried. Educational level was divided into three categories: less than high school graduate, junior college graduate or equivalent, and college graduate or higher. Household income was categorized as less than 5 million yen, 5–10 million yen, and >10 million yen. Employment status was classified into full-time job and not full-time job.

### 2.3. Statistical Analyses

The data were analyzed from 1,420 men who provided complete information for study variables. All analyses were stratified by BMI. Forced-entry adjusted logistic regression for gender, age, marital status, educational level, household income, and employment status was conducted to examine the association between environmental factors and meeting the PA recommendation. Adjusted odd ratios (ORs) and 95% confidence intervals (CI) were calculated for each variable. Likelihood ratio tests were used to compare models with or without interaction terms between environmental variables and BMI status. Inferential statistics were performed using SPSS 15.0, and the level of significance was set at *p* < 0.05.

## 3. Results

### 3.1. The Characteristics of the Participants

Table 1 presents the basic characteristics of the participants (mean age was 44.4 ± 8.3 years). Of all respondents, 31.1% were overweight, 70.4% were married, 64.4% had an education level of 4-year college/graduate school, 92.0% had full-time jobs, and 49.7% had a household income between 5,000,000 yen and 10,000,000 yen. The prevalence of achieving the PA recommendation (the sum of walking and other MVPA times) was 57.4% in the present study.

### 3.2. Perceived Environmental Factors Associated with Walking and MVPA (Excluding Walking) among Men

Table 2 shows the results of the adjusted logistic regression analysis in walking and MVPA (excluding walking) among normal-weight and overweight men. Ten significant environmental correlates of walking in normal-weight men and three in overweight men were observed. For normal-weight men, good access to shops (OR = 1.61; 95% CI: 1.24–2.10), good access to public transport (OR = 2.30; 95% CI: 1.57–3.38), good access to recreational facilities (OR = 1.42; 95% CI: 1.09–1.84), seeing people being active (OR = 1.49; 95% CI: 1.15–1.94), aesthetics (OR = 1.74; 95% CI: 1.33–2.29), street connectivity (OR = 1.48; 95% CI: 1.11–1.98), good maintenance of sidewalks (OR = 1.49; 95% CI: 1.14–1.94), good maintenance of bike lanes (OR = 1.58; 95% CI: 1.22–2.04), and presence of destination (OR = 1.61; 95% CI: 1.24–2.10) were significantly associated with engaging in 150 minutes of walking per week. However, having household cars or auto bikes (OR = 0.60; 95% CI: 0.41–0.88) was inversely associated with walking in normal-weight men. For overweight men, environmental factors associated with engaging in 150 minutes of walking per week were good access to recreational facilities (OR = 1.75; 95% CI: 1.18–2.58) and presence of destination (OR = 1.63; 95% CI: 1.10–2.41). Furthermore, lack of safety from crime during the day (OR = 0.48; 95% CI: 0.24–0.94) was negatively related to engagement in 150 minutes of walking per week.

Forced-entry, adjusted logistic regression analyses also indicated that connectivity of streets (OR = 1.45; 95% CI: 1.04–2.03) was a positive environmental factor associated with engaging in MVPA (excluding walking) for 150 minutes or more per week for normal-weight men. On the other hand, seeing people being active (OR = 2.27; CI: 1.38–3.75) was positively associated with engaging in MVPA (excluding walking) at the recommended level for overweight men.

Furthermore, significant interactions regarding walking were observed between BMI status and 2 environmental correlates: access to public transport (*P* = 0.03) and crime safety during the day (*P* = 0.01) (Table 3).

## 4. Discussion

In the present study, the perceived environmental attributes were significantly associated with PA among normal-weight and overweight Japanese men. The most important finding of the present study was that common environmental correlates of PA were observed between normal-weight and overweight men. Three environmental factors, good access to recreational facilities, seeing people being active, and presence of destination, were positively associated with meeting PA recommendation by either walking or MVPA (excluding walking). The results suggested that increasing the mix of utilitarian destination, supportive environment for seeing people being active, and convenience of accessing recreational facilities could encourage both normal-weight and overweight men to engage in sufficient PA for different purposes. In addition, these factors have been consistently revealed as environmental features related to PA among general populations in both Western countries and Japan [20,23,36–38]; this might strengthen the evidence for some common environmental features associated with PA among countries with different cultures and environments.

Conversely, access to public transport and safety from crime during the day were revealed as different environmental correlates of PA between normal-weight and overweight men based on likelihood ratio tests. This finding indicated that BMI status would be a potential moderator between the perceived environment and PA. Different environmental correlates of PA between socio-demographic subgroups have been examined in previous studies [22–26]. Different socio-demographic correlates of PA have also been reported among three BMI groups [27]. In addition, a previous study has observed that several perceived environmental factors (infrastructures, access to destinations, social environment and aesthetics) were associated with meeting the recommended PA level among overweight/obese women [22]. However, whether overweight men have different environmental correlates of PA than normal-weight men has not been discussed or analyzed as much as they have for women. A possible mechanism underlying the observed significance in perceived good access to public transport among normal-weight men alone is that overweight men are less likely to walk or cycle for transport in their daily lives than normal-weight men, regardless of the accessibility of public transport within their neighborhoods. Regarding the significant contribution of safety from crime only among overweight and obese men, they might be more sensitive to the presence of crime than normal-weight men because they may more easily experience discriminative and stigmatic treatment in their growing stage [39]. Therefore, the perception of an unsafe neighborhood environment might have a negative influence on their PA.

The findings of the present study suggest that consideration of not only general environmental correlates but also unique environmental correlates of PA among overweight and obese populations promote PA more effectively among these populations when environmental approaches for PA interventions are developed. One effective strategy for future environmental interventions aimed at increasing PA levels is promoting or changing their awareness of these environmental correlates. In addition, intervention approaches for rearranging or improving these environmental variables could be beneficial. For these approaches, it might be necessary to establish partnerships and collaborations with different sectors or organizations [40]. For example, neighborhood safety could be improved by cooperating with local authorities in organizing community groups to prevent crime. Furthermore, it could also be effective to cooperate with different government departments and non-government agencies (e.g., transportation department, local government, and transportation agencies) to adjust the location of public transport or number of services for transport-related walking.

The finding of the study indicated that the perceived environment-PA association was more related to normal-weight men than overweight men; while 11 perceived environmental factors associated with PA were found in normal-weight men, only four factors were significantly associated with PA in overweight men. This finding has not been reported in previous studies. Two studies have emphasized a stronger influence of perceived PA environment on older adults than on younger adults [24], as well as adults with disabilities than those without disabilities [41]. There are two implications of this finding. First, compared with normal-weight men, the environmental correlates of PA in overweight men were not detected well using IPAQ-E. As a result, objective measurements should be utilized to further examine the association between environmental factors and meeting the PA recommendation, especially on the walking behavior of overweight men. The second implication is that other factors (such as psychosocial correlates) might be more strongly associated with PA in overweight men than in normal-weight men. Thus, future studies are needed to identify the multiple levels of correlates associated with PA among normal-weight an overweight men.

In accordance with results from previous studies [18,33], the association of environmental factors from the IPAQ-E results were more related with walking than MVPA (excluding walking) between both normal-weight and overweight men in the present study. These results implied that walking behavior might be influenced more by the neighborhood environment than other types of PA behaviors. For future studies, it might be important to examine other correlates of specific MVPA behaviors.

For overweight men, seeing people being active was the strongest perceived environmental factor positively associated with engaging in 150 minutes of MVPA (excluding walking) per week. In previous studies, seeing people being active has been reported as a positive environmental correlate of being physically active [22,36]. The implication of the result is that overweight groups may need more social support to engage in MVPA (excluding walking), such as leisure-time PA, sports, and recreational activity [42–44].

Some limitations of the current study should be considered. First, the study had a cross-sectional design, making it impossible to determine causality. Second, the main measurements, which included BMI, environmental factors, and PA, were measured only by self-administrated questionnaires and could be subject to bias. The self-reported results may cause an underestimation of weight status [22] and an inaccurate estimation of PA time due to recall bias. Finally, the study has a limited ability to obtain representative samples because it relies on an internet-based survey. The respondents of internet-based surveys might have characteristics, such as younger, more educated, higher-income, having greater access to the internet, and more likely to respond to a survey, if they are interested in its contents or are attracted by the incentives offered for participation [45,46]. Thus, the results in the present study may be less applicable to those who have received less education and not applicable to the general population.

## 5. Conclusions

Both common and different environmental correlates of PA were observed among normal-weight and overweight men. The findings of the current study contribute evidence to the literature on moderators between environmental factors and PA. Findings from the present study suggested that developing different environmental intervention approaches might be needed to promote PA effectively for overweight populations compared with normal-weight populations. In addition, compared with normal-weight men, the perceived environmental correlates of PA in overweight men were not well defined. Future studies should consider examining multiple levels of correlates associated with different kinds of PA by utilizing both perceived and objective measurements among men with different BMI statuses.

## Figures and Tables

**Table 1.** Basic characteristics of all respondents stratified by normal-weight and overweight men.

	**Total sample N = 1,420**		**Normal weight N = 979 (68.9%)**		**Overweight N = 441 (31.1%)**			

	**N**	**%**	**N**	**%**	**N**	**%**	**X^2^**	***p***
**Age group**							3.43	0.18
30–39	475	33.4	338	34.5	137	31.1		
40–49	474	33.4	312	31.9	162	36.7		
50–59	471	33.2	329	33.6	142	32.2		
Mean age (± SD)	44.4 ± 8.3							
**Marital status**							0.03	0.86
Married	1,000	70.4	688	70.3	312	70.7		
Unmarried	420	29.6	291	29.7	129	29.3		
**Educational level**							2.04	0.36
Junior high/high school	330	23.2	219	22.4	111	25.2		
2-year college	176	12.4	118	12.1	58	13.2		
4-year college/graduate school	914	64.4	642	65.5	272	61.6		
**Job status**							0.09	0.77
full-time job	1,306	92.0	899	91.8	407	92.3		
not full-time job	114	8.0	80	8.2	34	7.7		
**Household income**							2.46	0.65
<5,000,000 yen	488	34.4	343	35.0	145	32.9		
<10,000,000 yen	706	49.7	481	49.1	225	51.0		
>10,000,000 yen	226	15.9	155	15.9	71	16.1		

SD = standard deviation.

**Table 2.** Adjusted model of perceived environmental factors associated with walking and MVPA (excluding walking) among normal-weight and overweight men.

	**Normal weight (N = 979, 68.9%)**				**Overweight (N = 441, 31.1%)**			

			**Walking**	**MVPA (excluding walking)**			**Walking**	**MVPA (excluding walking)**

	**N**	**%**	**Adjusted OR (95% CI)**	**Adjusted OR (95% CI)**	**N**	**%**	**Adjusted OR (95% CI)**	**Adjusted OR (95% CI)**
**Residential density**								
High	432	44.1	1.15 (0.89–1.50)	0.77 (0.57–1.03)	180	40.8	1.39 (0.94–2.07)	0.80 (0.49–1.29)
Low	547	55.9	1.00	1.00	261	59.2	1.00	1.00
**Access to shops**								
Good	553	56.5	1.61 (1.24–2.10)*	1.21 (0.90–1.63)	256	58.0	1.15 (0.78–1.70)	1.31 (0.81–2.11)
Poor	426	43.5	1.00*	1.00	185	42.0	1.00	1.00
**Access to public transport**								
Good	817	83.5	2.30 (1.57–3.38)*	1.23 (0.82–1.84)	360	81.6	1.17 (0.71–1.91)	1.28 (0.69–2.37)
Poor	162	16.5	1.00*	1.00	81	18.4	1.00	1.00
**Presence of sidewalks**								
Yes	604	61.7	1.29 (0.98–1.68)	1.04 (0.77–1.40)	267	60.5	1.43 (0.96–2.12)	0.93 (0.58–1.49)
No	375	38.3	1.00	1.00	174	39.5	1.00	1.00
**Presence of bike lanes**								
Yes	242	24.7	1.12 (0.83–1.51)	1.09 (0.78–1.52)	127	28.8	1.30 (0.85–1.99)	0.74 (0.43–1.26)
No	737	75.3	1.00	1.00	314	71.2	1.00	1.00
**Access to recreational facilities**								
Good	482	49.2	1.42 (1.09–1.84)*	1.29 (0.96–1.72)	221	50.1	1.75 (1.18–2.58)*	1.54 (0.96–2.47)
Poor	497	50.8	1.00*	1.00	220	49.9	1.00*	1.00
**Crime safety at night**								
Not safe	237	24.2	0.87 (0.64–1.17)	1.07 (0.77–1.49)	116	26.3	0.80 (0.52–1.25)	1.17 (0.70–1.95)
Safe	742	75.8	1.00	1.00	325	73.7	1.00	1.00
**Traffic safety**								
Not safe	354	36.2	1.16 (0.89–1.51)	1.03 (0.77–1.39)	159	36.1	1.06 (0.71–1.58)	1.20 (0.74–1.93)
Safe	625	63.8	1.00	1.00	282	63.9	1.00	1.00
**Seeing people being active**								
Yes	535	54.6	1.49 (1.15–1.94)*	1.32 (0.98–1.77)	250	56.7	1.41 (0.95–2.09)	2.27 (1.38–3.75)**
No	444	45.4	1.00*	1.00	191	43.3	1.00	1.00**
**Aesthetics**								
Yes	351	35.9	1.74 (1.33–2.29)*	1.29 (0.96–1.74)	149	33.8	1.14 (0.76–1.71)	1.28 (0.79–2.07)
No	628	64.1	1.00*	1.00	292	66.2	1.00	1.00
**Connectivity of streets**								
Yes	700	71.5	1.48 (1.11–1.98)*	1.45 (1.04–2.03)**	321	72.8	1.05 (0.68–1.62)	0.79 (0.48–1.32)
No	279	28.5	1.00*	1.00**	120	27.2	1.00	1.00
**Maintenance of sidewalks**								
Good	555	56.7	1.49 (1.14–1.94)*	1.10 (0.82–1.47)	256	58.0	1.11 (0.75–1.64)	0.82 (0.51–1.30)
Poor	424	43.3	1.00*	1.00	185	42.0	1.00	1.00
**Maintenance of bike lanes**								
Good	479	48.9	1.58 (1.22–2.04)*	1.14 (0.85–1.52)	216	49.0	1.01 (0.69–1.48)	0.90 (0.57–1.43)
Poor	500	51.1	1.00*	1.00	225	51.0	1.00	1.00
**Traffic safety for bicyclists**								
Not safe	427	43.6	0.96 (0.74–1.24)	0.89 (0.67–1.19)	192	43.5	1.16 (0.79–1.71)	0.92 (0.57–1.47)
Safe	552	56.4	1.00	1.00	249	56.5	1.00	1.00
**Crime safety during the day**								
Not safe	106	10.8	1.45 (0.96–2.18)	1.10 (0.69–1.74)	46	10.4	0.48 (0.24–0.94)*	0.88 (0.41–1.92)
Safe	873	89.2	1.00	1.00	395	89.6	1.00*	1.00
**Presence of destination**								
Yes	511	52.2	1.61 (1.24–2.10)*	1.12 (0.83–1.50)	247	56.0	1.63 (1.10–2.41)*	1.22 (0.76–1.96)
No	468	47.8	1.00*	1.00	194	44.0	1.00*	1.00
**Household car or auto bikes**								
One or more	845	86.3	0.60 (0.41–0.88)*	1.43 (0.91–2.26)	394	89.3	0.54 (0.28–1.02)	1.56 (0.66–3.69)
None	134	13.7	1.00*	1.00	47	10.7	1.00	1.00

Adjusted for age, marital status, educational level, household income, and employment status.

*, ** statistically significant (*p* < 0.05).

**Table 3.** Significance of interactions between BMI status and environmental variables by binary logistic regression models.

	***P* value for interaction term with BMI status**	
	**Walking**	**MVPA (excluding walking)**

	***P* value**	***P* value**
**Residential density (High)**	0.46	0.66
**Access to shops (Good)**	0.16	0.83
**Access to public transport (Good)**	0.03**	0.94
**Presence of sidewalks (Yes)**	0.75	0.60
**Presence of bike lanes (Yes)**	0.67	0.19
**Access to recreational facilities (Good)**	0.31	0.52
**Crime safety at night (Safe)**	0.85	0.73
**Traffic safety (Safe)**	0.65	0.55
**Seeing people being active (Yes)**	0.76	0.14
**Aesthetics (Yes)**	0.08	0.70
**Connectivity of streets (Yes)**	0.18	0.06
**Maintenance of sidewalks (Good)**	0.22	0.28
**Maintenance of bike lanes (Good)**	0.06	0.40
**Traffic safety for bicyclists (Safe)**	0.39	0.76
**Crime safety during the day (Safe)**	0.01**	0.69
**Presence of destination (Yes)**	0.99	0.75
**Household car or auto bikes (One or more)**	0.93	0.66

Adjusted by age, marital status, educational level, household income, employment status and BMI status.

** statistically significant (*p* < 0.05).
